# Complete Genome Sequence of *Nectarine stem pitting-associated virus*, Isolated from *Prunus persica* in Cheongdo County, South Korea

**DOI:** 10.1128/genomeA.00908-17

**Published:** 2017-08-31

**Authors:** Davaajargal Igori, Dasom Baek, San Yeong Kim, Euncheol Seo, Su-Heon Lee, Rae-Dong Jeong, Seung-In Yi, Jin-Jong Bong, Jae Sun Moon

**Affiliations:** a Biotechnology Program, University of Science and Technology, Daejeon, Republic of Korea; b Plant Systems Engineering Research Center, Korea Research Institute of Bioscience and Biotechnology, Daejeon, Republic of Korea; c Cheongdo Peach Research Institute, Gyeongsangbuk-do Agricultural Research and Extension Services, Cheongdo, Republic of Korea; d School of Applied Biosciences, Kyungpook National University, Daegu, Republic of Korea; e Department of Applied Biology, Chonnam National University, Gwangju, Republic of Korea; f Korea Seed and Variety Service, Ministry of Agriculture, Food and Rural Affairs, Gimcheon-si, Republic of Korea; g NEXBIO Co., Ltd., Daejeon, Republic of Korea

## Abstract

We report here the first complete genome sequence of a South Korean isolate of *Nectarine stem pitting-associated virus* (NSPaV) from peach and compare it with previously described complete NSPaV genome sequences. The highest whole-genome nucleotide sequence identity was 95.3% with GenBank accession no. KT273409 (NSPaV) from the United States.

## GENOME ANNOUNCEMENT

*Nectarine stem pitting-associated virus* (NSPaV) is a single-stranded positive-sense RNA virus of the genus *Luteovirus* in the family *Luteoviridae*, which was first described in the United States in nectarine (*Prunus persica*) (1, 2). It was recently detected in *Prunus* spp. in China (3), Japan (4), South Korea (5), and Hungary (6). The NSPaV genome has four open reading frames.

Peach (genus *Prunus*, family *Rosaceae*) is an economically important crop that is infected by a number of plant viruses and viroids. Peach tree (*Prunus persica*) leaves with and without symptoms were collected in Cheongdo County, South Korea, in May 2015. Total RNA was isolated from the leaf tissue using a WizPrep Plant RNA minikit (Wizbiosolutions, Seongnam, South Korea). Before library construction, ribosomal RNA (rRNA) was removed from extracted total RNA using a Ribo-Zero rRNA removal kit (Plant Leaf; Epicentre, Madison, WI, USA). A library was constructed from the rRNA-depleted RNA using a TruSeq RNA sample prep kit (Illumina, San Diego, CA, USA) and sequenced using an Illumina HiSeq2500 sequencer. The obtained raw reads were trimmed and then *de novo* assembled from the remaining high-quality reads using Trinity software. BLASTn analysis showed that, among the assembled contigs, one long contig, representing nearly the entire genome (4,874 bp), had the highest nucleotide sequence similarity (95% identity) to NSPaV (GenBank accession no. KT273409) and was designated the NSPaV-SK contig. To confirm the sequence of the NSPaV-SK contig, specific primer sets were designed based on the contig sequence. Complementary DNA (cDNA) was synthesized from isolated total RNA from a peach sample using a random N25 primer with RevertAid reverse transcriptase (RT) (Thermo Scientific, Waltham, MA, USA). RT-PCR was conducted with the synthesized cDNA and specific primer pairs using AccuPower ProFi *Taq* PCR premix (Bioneer, Daejeon, South Korea). RT-PCR products were cloned into the RBC T&A cloning vector (RBC Bioscience, Taipei, Taiwan) and sequenced by GenoTech (Daejeon, South Korea). To complete the genome sequence of NSPaV-SK, the 5′ and 3′ termini of the viral RNA were verified using a 5′/3′ RACE system for the rapid amplification of cDNA (Invitrogen, Carlsbad, CA, USA) (7). All amplified fragments were cloned, sequenced, and assembled using the DNAman version 5.2.10 program. The sequence constituted the first complete genome sequence of a South Korean isolate of NSPaV, containing 4,991 nucleotides (nt), with a 5′ untranslated region (UTR) of 131 nt and a 3′ UTR of 671 nt (accession no. MF326520). Upon pairwise comparison analysis, the highest nucleotide identity for the complete genome was 95.3% to KT273409 (NSPaV) from the United States. NSPaV-SK had the highest amino acid similarities with KT273409 at P1 (92.1%) and P1 to P2 (96.2%) and with KT273410 at P3 (97.6%) and P3 to P5 (96.6%). Here, we report the first full-length genome sequence of a South Korean isolate of NSPaV.

### Accession number(s).

The complete genome sequence of the South Korean isolate NSPaV-SK has been deposited in GenBank under the accession number MF326520.
